# *Ananas comosus* L. Leaf Phenols and p-Coumaric Acid Regulate Liver Fat Metabolism by Upregulating CPT-1 Expression

**DOI:** 10.1155/2014/903258

**Published:** 2014-08-12

**Authors:** Weidong Xie, Shaobo Zhang, Fan Lei, Xiaoxi Ouyang, Lijun Du

**Affiliations:** ^1^Division of Life Science & Health, Graduate School at Shenzhen, Tsinghua University, Shenzhen 518055, China; ^2^Zhu Jiang Hospital, Southern Medical University, Guangzhou 510282, China; ^3^Protein Science Laboratory of the Ministry of Education, Laboratory of Pharmaceutical Science, School of Life Science, School of Medicine, Tsinghua University, Beijing 100084, China

## Abstract

In this study, we aimed to investigate the effect and action mechanisms of pineapple leaf phenols (PLPs) on liver fat metabolism in high-fat diet-fed mice. Results show that PLP significantly reduced abdominal fat and liver lipid accumulation in high-fat diet-fed mice. The effects of PLP were comparable with those of FB. Furthermore, at the protein level, PLP upregulated the expression of carnitine palmitoyltransferase 1 (CPT-1), whereas FB had no effects on CPT-1 compared with the HFD controls. Regarding mRNA expression, PLP mainly promoted the expression of CPT-1, PGC1a, UCP-1, and AMPK in the mitochondria, whereas FB mostly enhanced the expression of Ech1, Acox1, Acaa1, and Ehhadh in peroxisomes. PLP seemed to enhance fat metabolism in the mitochondria, whereas FB mainly exerted the effect in peroxisomes. In addition, p-coumaric acid (CA), one of the main components from PLP, significantly inhibited fat accumulation in oleic acid-induced HepG2 cells. CA also significantly upregulated CPT-1 mRNA and protein expressions in HepG2 cells. We, firstly, found that PLP enhanced liver fat metabolism by upregulating CPT-1 expression in the mitochondria and might be promising in treatment of fatty liver diseases as alternative natural products. CA may be one of the active components of PLP.

## 1. Introduction

The prevalence of nonalcoholic fatty liver diseases (NAFLDs) is about 20% in mainland China and even higher in developed countries or areas [1]. NAFLDs exhibit adverse levels of liver fibrosis and cardiometabolic risk factors [2]. Besides genetic factors, environmental factors, such as high-fat diets, have an important function in the development of NAFLDs [3]. NAFLDs are considered to be diseases of affluence [4].

The management of NAFLDs still faces a great challenge [5]. Fibrates exert lipid-lowering effects on the blood and liver by targeting peroxisomal proliferator-activated receptor alpha (PPAR*α*) and promoting fat metabolism in the liver [6]. However, the effects of fibrates on liver histology are minimal [7]. Statins lower cholesterol synthesis in the liver by inhibiting HMGCoA reductase, and their uses in patients with hyperlipidemia and NAFLD are justified; however, neither of the trials reported possible histological changes in NAFLD when subjected to statin therapy [8]. Current drugs used for the treatment of NAFLDs require further improvement.

Pineapple (*Ananas comosus *L.) is grown largely in Hawaii, the Philippines, Caribbean area, Malaysia, Taiwan, Thailand, Australia, Mexico, Kenya, South Africa, and southern China. Pineapple has various agricultural utilities, such as fruits for nutrition, and some folk medicinal uses have been found. Pineapple leaves can help in digestion [9]. Pineapple leaf phenols (PLPs) demonstrated hypolipidemic effects in previous studies [10, 11]. PLPs have different hypolipidemic mechanisms from fenofibrate (FB). However, whether PLPs have an effect on NAFLDs remains unclear. Moreover, the molecular mechanisms of PLP are unknown.

Carnitine palmitoyltransferase 1 (CPT-1) is a mitochondrial membrane protein that converts long-chain fatty acyl-CoA molecules to their corresponding acylcarnitine molecules [12]. CPT-1 deficiency causes a disorder of long-chain fatty acid oxidation [13]. The upregulation of CPT-1 is associated with the inhibition of fatty liver formation [14]. Some plant phenols have been reported to increase CPT-1 expression and attenuate hepatic steatosis [15].

In this study, we investigated the effect of PLP on the formation of fatty livers in high-fat diet-fed mice and found that PLP inhibited liver fat accumulation by regulating CPT-1 expression.

## 2. Materials and Methods

### 2.1. Animals and Diets

Three-week-old male NIH mice were purchased from Guangdong Medical Animal Center (Guangzhou, China). The animals were housed in an environmentally controlled animal room (temperature: 20°C ± 2°C; humidity: 60% ± 5%; and a 12 h dark/light cycle). Animals were fed chow diets and water* ad libitum*. The study was strictly carried out in accordance with the recommendations in the Guide for the Care and Use of Laboratory Animals of Institutional Animal Care and Use Committee of Tsinghua University. The protocol was approved by the Animal Welfare and Ethics Committee of Tsinghua University, China (2013-XWD-BC). Both normal chow and high-fat diets were purchased from Shanghai SLAC Laboratory Animal Co., Ltd. Normal chow diets contained 12% calories in fat while high-fat diets contained 38% calories in fat.

### 2.2. Drugs

Pineapple leaves were collected from Boao, Hainan, China. PLPs (batch number 051201) were prepared as previously prescribed [11]. Phytochemical assays showed that PLP mainly contained total phenols (more than 60%, w/w, in terms of the extract). p-coumaric acid (CA) was a compound separated from PLPs. PLP contained 1.5% CA (w/w). HPLC figures of PLP can be tracked from the previous study [11]. FB was purchased from Sigma-Aldrich. Drugs were freshly prepared before administration.

### 2.3. Experimental Procedure

Male NIH mice were divided into four groups, namely, normal control mice (normal), high-fat diet-fed control mice (HFD), and PLP- and FB-treated high-fat diet-fed groups (PLP and FB, resp.). First, mice were fed high-fat diets (HFD, PLP, and FB groups) or normal chow diets (normal group) for four weeks. PLP and FB were orally administered once daily at 300 and 200 mg/kg, respectively. The dosages of PLP and FB were selected according to a previous report (200–800 mg/kg) [11] with a slight modification. The drugs were suspended with distilled water. Normal and HFD control mice were treated with identical volumes of distilled water. Diet intake was periodically measured in metabolic cages within 24 h. After four weeks of administration, the mice were weighed. Livers and abdominal adipose tissues were removed and weighed. Parts of the liver tissues were stored at −80°C or soaked in 10% formalin solution for further biochemical or histopathology assays, respectively.

### 2.4. Biochemical Assays

Liver fat assays were performed according to a previous protocol with slight modification [16]. In brief, five mice from each group were randomly selected. About 35 ± 5 mg of liver from each mouse was weighed precisely and homogenized with 0.2 mL of PBS plus 1 mL of chloroform-methanol mixture solution (CHCl_3_-CH_3_OH; 2 : 1, v/v) in an Eppendorf tube. The extraction solution was centrifuged at 10,000 ×g for 5 min. A 20 (for triglyceride assay) and 40 *μ*L (for cholesterol assay) aliquot of the lower phase were added to another Eppendorf tube and air-dried at 37°C, respectively. The dried lipids at the bottom of the tubes were used to assay triglycerides and cholesterol levels in livers. Triglycerides and cholesterols in livers were assayed by using triglyceride and cholesterol reagent kits (BioSino Biotechnology and Science Inc., Beijing, China) according to the GPO-PAP and CHOD-PAP calorimetric methods, respectively. Briefly, the dried lipids in the bottom of the tubes were incubated with 200 *μ*L of triglyceride or cholesterol reagents at 37°C for 30–60 min. Then, the reaction liquids were transferred into a 96-well plate and measured at 500 nm through a Microplate Reader (Thermo Scientific Varioskan Flash). The lipid contents were normalized with liver weight, and data were expressed as lipids/liver weight (mg/g).

### 2.5. Histopathology

Five mice from each group were randomly selected. A liver tissue mass (2 mm diameter) from each mouse was fixed with 10% formalin and processed for routine paraffin-wax histology. Sections were stained with hematoxylin and eosin (H&E). Five continuous slices (thickness of 10 *μ*m; gap of 20 *μ*m) were obtained to measure the formation of fat droplets in the liver of each mouse. We transferred the H&E pictures into black and white ones. Fat droplets formed in liver sections could be transferred into white, bright areas while other liver tissue areas' background became black by adjusting the contrast and brightness appropriately. These grey density values of white, bright areas from the liver fat droplets can be calculated by Gel-Pro software (Media Cybernetics, USA) and subjected to further statistical analysis.

### 2.6. mRNA Analysis by Quantitative Real-Time PCR (qPCR)

Five mice from each group were randomly selected. Total RNA of liver tissues was extracted using RNAiso plus reagent (Takara, Dalian, China) according to the manufacturer's instructions. Reverse transcriptase (RT) was performed using a kit containing reverse transcriptase M-MLV (RNase H^−^, code number D2639A, Takara, Dalian, China) according to the manufacturer's instructions. RT was conducted using an Alpha Unit Block Assembly for DNA Engine systems (Bio-Rad, USA) with a thermocycler program consisting of cDNA synthesis at 42°C for 60 min, RNase inactivation at 70°C for 15 min, and sample cooling at 4°C for 10 min. Primers were synthesized from Invitrogen (Table 1). Actin was used as an internal control for normalization. qPCR analysis was conducted using SYBR Green I Real-Time PCR Master Mix following the manufacturer's protocol (Code number QPT-201, TOYOBO, Osaka, Japan) in an ABI PRISM 7300 Real-Time PCR System (Applied Biosystems, USA). qPCR analysis was performed in two steps. First, cDNA samples were predenatured at 95°C for 60 s. Second, denatured cDNA samples were amplified with 40 cycles at 95°C for 15 s, 60°C for 15 s, and 72°C for 45 s. Data were analyzed by the raw relative quantitation method (2^−ddCt^).

### 2.7. Western Blot

Five mice from each group were randomly selected. Liver tissues were homogenized and lysed with NETN buffer, and lysates were centrifuged at 10,000 ×g at 4°C for 10 min. Supernatants were collected, and the protein concentration was determined using a BCA assay kit (Nanjing Jiancheng Biotech, China). Western blot analysis was carried out according to the manufacturer's protocol. Goat polyclonal antibody against CPT-1A of human origin (also recommended for the detection of mouse origin, Santa Cruz; 1 : 500–1000 dilution), rabbit polyclonal antibody against enoyl coenzyme A hydratase 1, peroxisomal (Ech1) of human origin (also recommended for the detection of mouse origin, Beijing Aviva Systems Biology, China; 1 : 500–1000 dilution), and mouse monoclonal antibody against beta-actin of chicken origin (also recommended for the detection of mouse origin, Santa Cruz; 1 : 500–1000 dilution) were used. Protein expression was visualized with secondary antibodies (donkey versus goat, goat versus rabbit, and rabbit versus mouse IgG-HRP; Amersham Biosciences, USA; 1 : 2000) and enhanced chemiluminescence (KPL, USA). Grey density values of Ech1 and CPT-1A protein expression were normalized with beta-actin (reference protein).

### 2.8. Oleic Acid-Induced HepG2 Cells

HepG2 cells at a density of 5 × 10^4^/well (2 mL of cell culture medium) were placed in a 24-well plate and cultured in fresh medium (DMEM + 10% FBS, GIBCO). Oleic acid (Sigma-Aldrich, St. Louis, MO, USA) was added to the bovine serum albumin- (BSA-) phosphate buffer saline (PBS) mixture in 2 : 1 molar ratio (Oleic acid : BSA) and formed a BSA-coupled oleic acid solution. After 12 h of incubation, BSA-coupled oleic acid solution at the final concentration of 0.6 *μ*M was added to cell mediums and used to induce the formation of fatty liver model* in vitro*. An identical volume of BSA-PBS solution was used as blank control for BSA-coupled oleic acid solution. Simultaneously, PLP (final concentrations of 10 and 50 *μ*g/mL, resp.), CA (final concentrations of 1 and 10 *μ*g/mL, resp.; Sigma-Aldrich), and FB (final concentration of 1.4 *μ*g/mL) were dissolved in dimethyl sulfoxide (DMSO) and added to the cell medium. These dosages were not toxic to cells. An identical volume of DMSO served as the control for tested drugs. After 24 h, the cells were washed with PBS twice and fixed with 4% paraformaldehyde in PBS for 20 min. The cells were then washed with PBS twice and stained with 0.3% oil red O solution (dissolved in 60% isopropanol/water, v/v) for 30 min. The stained cells were washed with PBS three times, and the formation of lipid droplets in HepG2 cells was observed. Isopropanol was used to dissolve the oil red O stain in the cells, and the optical density was assayed at 540 nm. The assay was performed in triplicate for each sample.

### 2.9. Immunofluorescence Assay in HepG2 Cells

First, circular transparent glass slides (10 mm diameter) were placed at the bottom of a six-well plate. HepG2 cells at a density of 2.5 × 10^5^/well (2 mL of cell culture medium) were added to the surface of glass slides in a six-well plate. Some cells were immobilized into the slides. The cells were cultured with fresh medium (DMEM + 10% FBS). After 12 h, PLP (final concentrations of 10 and 50 *μ*g/mL, resp.), CA (final concentrations of 1 and 10 *μ*g/mL, resp.), and FB (final concentration of 1.4 *μ*g/mL) were dissolved in DMSO and added to the cell medium, respectively. An identical volume of DMSO served as the control. After 24 h, the immobilized cells in the slides were washed with PBS and fixed with 4% paraformaldehyde in PBS. The cells were then washed with PBS three times and incubated with 0.1% triton in PBS for 15 min. After three washes with PBS, the cells were blocked with 3% bovine serum albumin in PBS for 1 h. The cells were incubated with goat polyclonal antibody against CPT-1A of human origin (also recommended for the detection of mouse origin, 1 : 100, Santa Cruz) in PBS for 1 h and washed with PBS three times. The cells were then incubated with donkey anti-goat IgG H&L (Alexa Fluor 647, 1 : 100, Abcam) in PBS for 1 h. Fluorescence signals of the cells were collected by confocal microscopy (OLYMPUS) and assayed by FV10-ASW Viewer 3.1 and Gel-Pro software.

### 2.10. RT-PCR and Western Blot Assays in HepG2 Cells

To validate the CPT-1 expression, we conducted RT-PCR and Western blot assays in HepG2 cells, respectively. The cells at a density of 2.5 × 10^5^/well (2 mL of cell culture medium) were cultured in 6-well plate with fresh medium (DMEM + 10% FBS). After 12 h, PLP (final concentrations of 10 and 50 *μ*g/mL, resp.), CA (final concentrations of 1 and 10 *μ*g/mL, resp.), and FB (final concentration of 1.4 *μ*g/mL) were dissolved in DMSO and added to the cell medium, respectively. An identical volume of DMSO served as the control. Simultaneously, the cells were incubated with oleic acid (final concentration of 0.6 *μ*M) to induce fatty liver model* in vitro*. In another separate trial, the cells were incubated only with the tested drugs and were not subjected to oleic acid treatment. After 24 h, the cells were collected for RT-PCR and Western blot assays. For RT-PCR, human CPT-1A (forward: ATCAATCGGACTCTGGAAACGG, reverse: TCAGGGAGTAGCGCATGGT), CPT-1B (forward: GCGCCCCTTGTTGGATGAT, reverse: CCACCATGACTTGAGCACCAG), and actin (forward: CATGTACGTTGCTATCCAGGC, reverse: CTCCTTAATGTCACGCACGAT) primers were synthesized from Invitrogen. For Western blot, we assayed the expressions of CPT-1A and actin. Actin was served as internal control for normalization. RT-PCR and Western blot were conducted as described above, respectively.

### 2.11. Statistical Analysis

Data are expressed as mean ± SD, and mean comparisons in groups were performed using one-way ANOVA. Tukey-Kramer comparisons were used to determine the source of significant differences. Differences with *P* < 0.05 were considered to be statistically significant.

## 3. Results

### 3.1. Body Weight Index and Diet Intake

**Figure 1:**
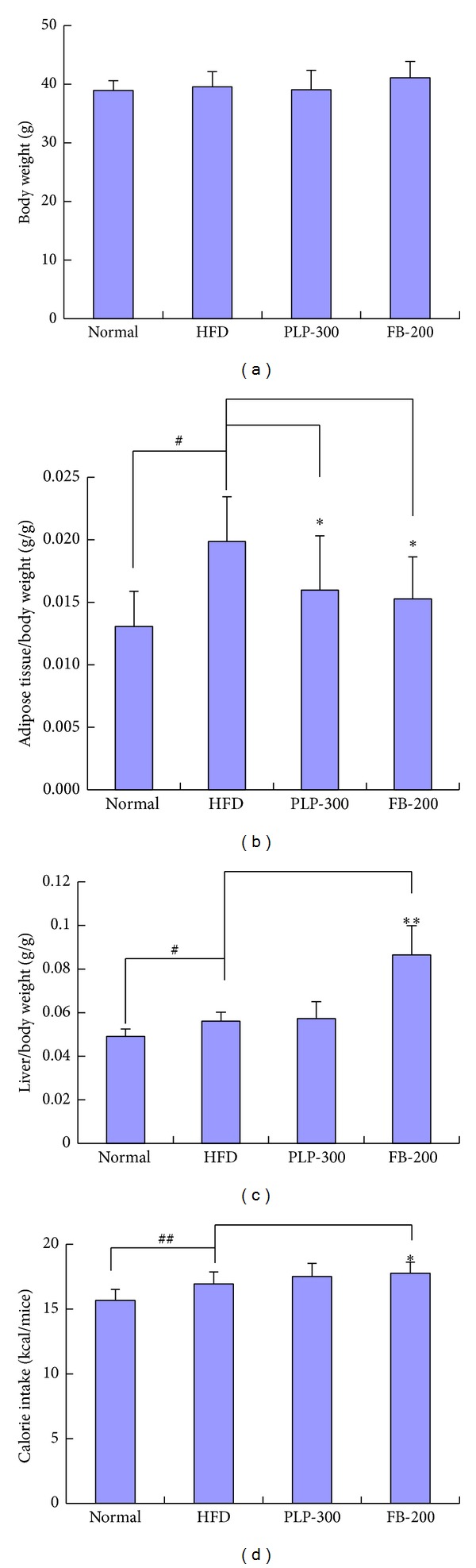
(a) Changes in body weight of mice; (b) adipose tissue/body weight of mice; (c) liver/body weight of mice; and (d) changes in dietary calories of mice within 24 h. Normal: normal control mice; HFD: high-fat diet-fed control mice; PLP-300: pineapple leaf phenol-treated (300 mg/kg) HFD mice; and FB-200: fenofibrate-treated (200 mg/kg) HFD mice. Data are expressed as mean ± SD (*n* = 10). ^#^ *P* < 0.05, ^##^ *P* < 0.01 versus normal controls; ∗*P* < 0.05, ∗∗*P* < 0.01 versus HFD controls.

The high-fat diet-fed mice showed no significant increase in body gain compared with the normal controls (Figure 1(a)). Both PLP and FB did not show significant effects on the high-fat diet-fed mice compared with the HFD controls. However, HFD mice showed higher abdominal fat accumulation than normal controls; increased fat accumulation in HFD mice was attenuated by both PLP and FB (Figure 1(b)). In addition, HFD mice showed a slight increase in liver weight than normal controls; PLP did not affect liver weight, but FB increased liver weight significantly (Figure 1(c)). HFD mice demonstrated a slight increase in dietary calorie intake compared with normal controls. PLP seemed to have no significant effect on calorie intake in HFD mice, but FB showed a slight increase in calorie intake in HFD mice compared with that in HFD controls (Figure 1(d)).

### 3.2. Fat Contents in the Livers

**Figure 2:**
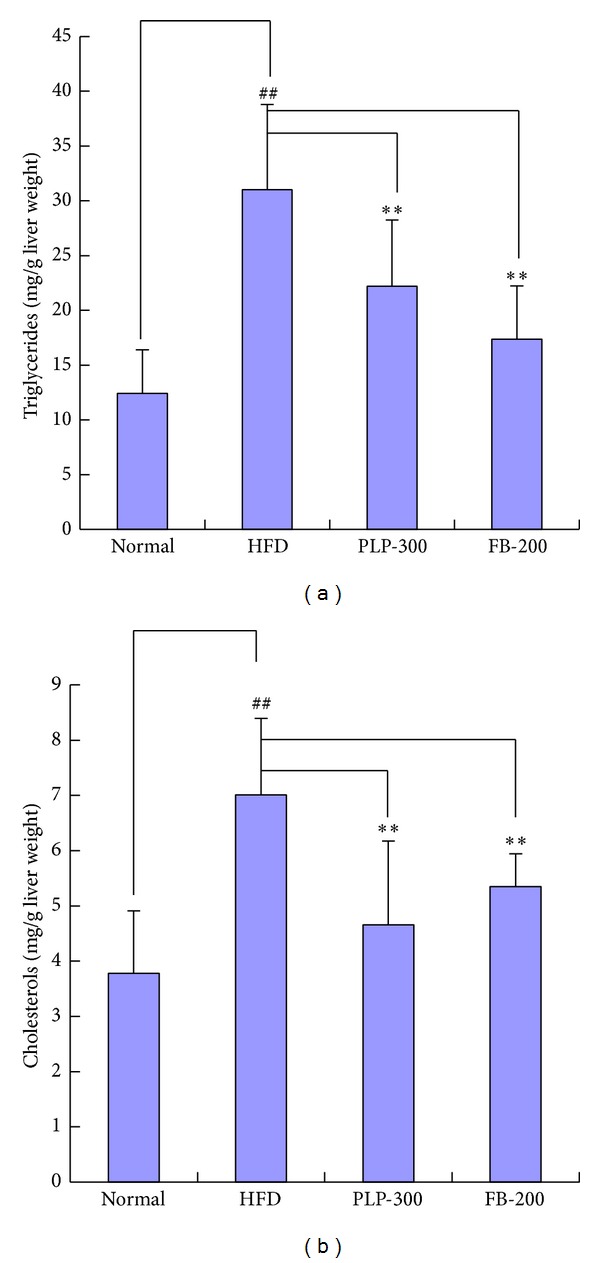
Lipid contents in the livers of mice. Normal: normal control mice; HFD: high-fat diet-fed control mice; PLP-300: pineapple leaf phenol-treated (300 mg/kg) HFD mice; and FB-200: fenofibrate-treated (200 mg/kg) HFD mice (*n* = 5). Data are expressed as mean ± SD (*n* = 5). ^##^ *P* < 0.01 versus normal controls and ∗∗*P* < 0.01 versus HFD controls.

Biochemical assays showed that the fat contents in livers of mice from the HFD group significantly increased, but this increase was attenuated by both PLP and FB. FB seemed to have a stronger effect than PLP (Figure 2). However, FB significantly increased the liver weights, as reported previously.

### 3.3. Liver Tissue Sections

**Figure 3:**
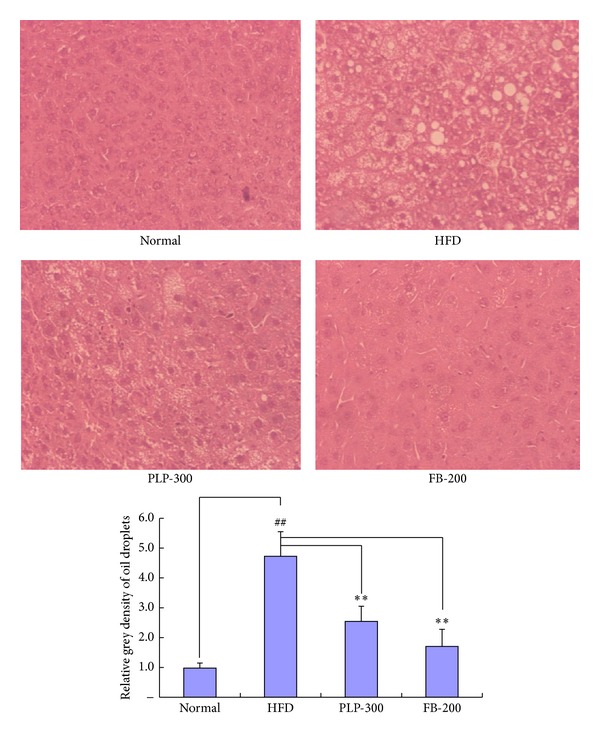
H&E staining images from liver sections of mice (200x). Normal: normal control mice; HFD: high-fat diet-fed control mice; PLP-300: pineapple leaf phenol-treated (300 mg/kg) HFD mice; and FB-200: fenofibrate-treated (200 mg/kg) HFD mice (*n* = 5). ^##^ *P* < 0.01 versus normal controls and ∗∗*P* < 0.01 versus HFD controls.

According to the liver histochemical sections stained with H&E, high-fat diet-fed mice showed a significant accumulation of fat droplets in the liver (Figure 3). However, PLP and FB significantly attenuated the formation of fat droplets in the liver. FB was more effective than PLP in inhibiting fat accumulation.

### 3.4. mRNA Expression Determined by qPCR

**Figure 4:**
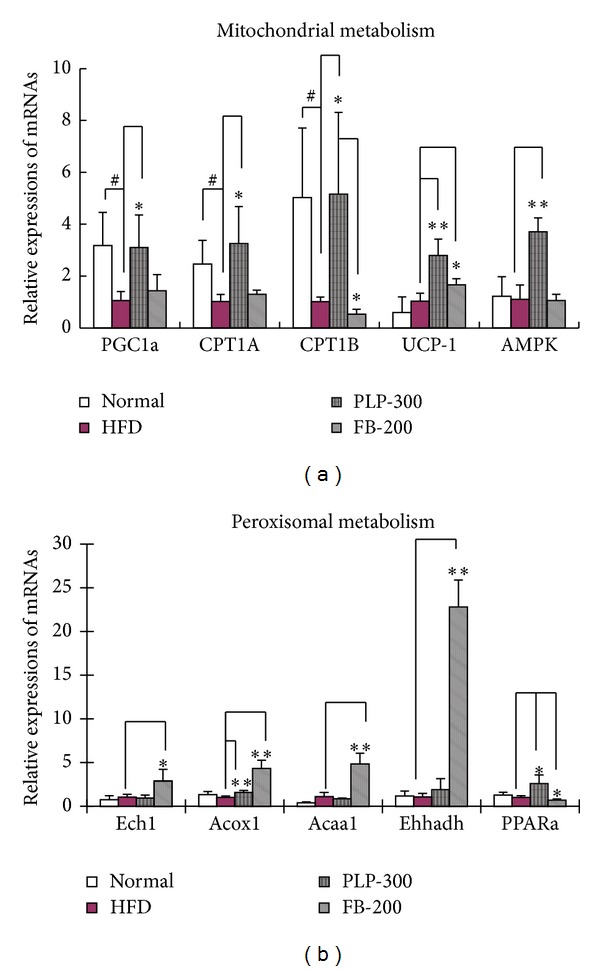
Expression of mitochondrial (a) and peroxisomal (b) genes in livers of mice, as determined by qPCR. Normal: normal control mice; HFD: high-fat diet-fed control mice; PLP-300: pineapple leaf phenol-treated (300 mg/kg) HFD mice; and FB-200: fenofibrate-treated (200 mg/kg) HFD mice. Data are expressed as mean ± SD (*n* = 5). ^#^ *P* < 0.05 versus normal controls and ∗*P* < 0.05, ∗∗*P* < 0.01 versus HFD controls.

PLP significantly upregulated the mRNA expression of CPT-1 (CPT-1A and CPT-1B), a key rate-limiting enzyme responsible for mitochondrial fat metabolism (Figure 4(a)). Also, PLP induced higher expression levels of mitochondrial uncoupling protein 1 (UCP-1), peroxisomal proliferator-activated receptor gamma coactivator *α* (PGC1*α*) and adenosine 5′-monophosphate- (AMP-) activated protein kinase (AMPK) than FB, which were associated with increased mitochondrial metabolism. However, FB showed more expressions of Ech1, acyl-CoA oxidase 1 palmitoyl (Acox1), 3-ketoacyl-CoA thiolase (Acaa1), and enoyl-CoA hydratase/*L*-3-hydroxyacyl-CoA dehydrogenase (Ehhadh) (Figure 4(b)), which is responsible for enhanced peroxisomal metabolism. In addition, PLP and FB had adverse effects on the expression of peroxisomal proliferator-activated receptor alpha (PPAR*α*) and CPT-1B. Taken together, PLP seemed to exert different effects on the expression of most genes compared with FB.

### 3.5. Protein Expression Determined by Western Blot

**Figure 5:**
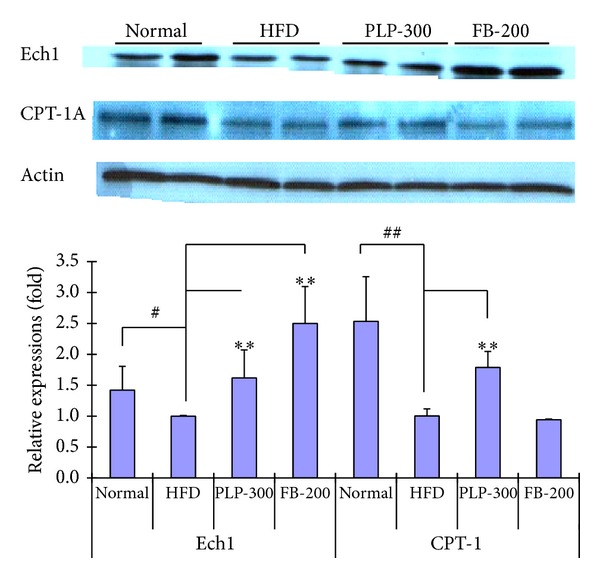
Expression of Ech1 and CPT-1A in livers of mice, as determined by Western blot. Normal: normal control mice; HFD: high-fat diet-fed control mice; PLP-300: pineapple leaf phenol-treated (300 mg/kg) HFD mice; and FB-200: fenofibrate-treated (200 mg/kg) HFD mice. Data are expressed as mean ± SD (*n* = 5). ^#^ *P* < 0.05, ^##^ *P* < 0.01 versus normal controls; ∗∗*P* < 0.01 versus HFD controls.

Ech1 was downregulated in high-fat diet-fed mice. However, PLP slightly upregulated Ech1 expression, whereas FB largely upregulated Ech1 expression (Figure 5). CPT-1A is one main subtype of CPT1 expressed in livers. Here, CPT-1A was also significantly downregulated in high-fat diet-fed mice. PLP significantly upregulated the expression of CPT-1A, whereas FB had no significant effect on CPT-1A expression. Thus, PLP may attenuate the formation of fatty livers by increasing CPT-1A expression. However, the compounds contributing to this effect remain unclear.

### 3.6. Effects of PLP and CA in Oleic Acid-Induced HepG2 Cells

**Figure 6:**
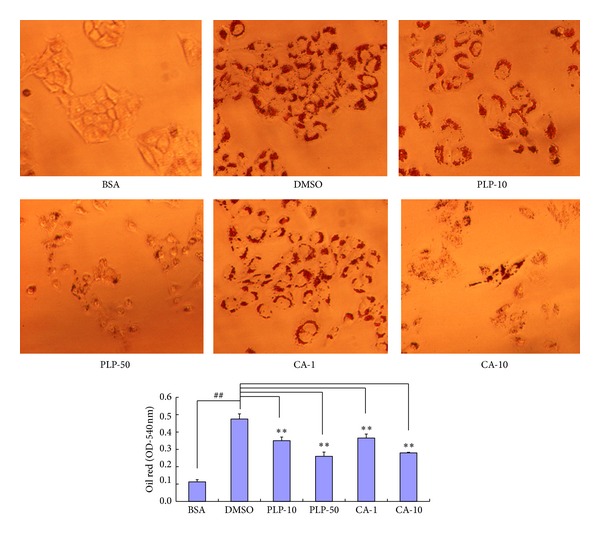
Effect of PLP and CA in oleic acid-induced HepG2 cells. BSA: bovine serum albumin-treated blank control; DMSO: DMSO-treated oleic acid-induced control cells; PLP-10 and PLP-50: pineapple leaf phenol-treated (final concentrations of 10 and 50 *μ*g/mL, resp.) cells; and CA-1 and CA-10: coumaric acid-treated (final concentrations of 1 and 10 *μ*g/mL, resp.) oleic acid-induced cells. Data are expressed as mean ± SD (*n* = 3). ^##^ *P* < 0.01 versus BSA controls; ∗∗*P* < 0.01 versus DMSO controls.

AC is one of the main components of PLPs [17] and demonstrates good pharmacokinetic behavior after oral administration [18]. In this study, we selected PLP and CA for further validation* in vitro*. Incubation of 0.6 *μ*M oleic acid for 24 h significantly increased fat accumulation in HepG2 cells (Figure 6). However, PLP (final concentrations of 10 and 50 *μ*g/mL, resp.) and CA (final concentrations of 1 and 10 *μ*g/mL, resp.) significantly attenuated fat accumulation in HepG2 cells. FB (final concentration of more than 10 *μ*g/mL) showed significant cell toxicity responses in HepG2 cells. Thus, a low concentration (1.4 *μ*g/mL) was used for further investigation. However, FB at 1.4 *μ*g/mL did not show a significant effect (data not shown). Nevertheless, these results show that CA could be an active component of PLP.

### 3.7. Effects of PLP and CA on CPT-1 Expression in HepG2 Cells

**Figure 7:**
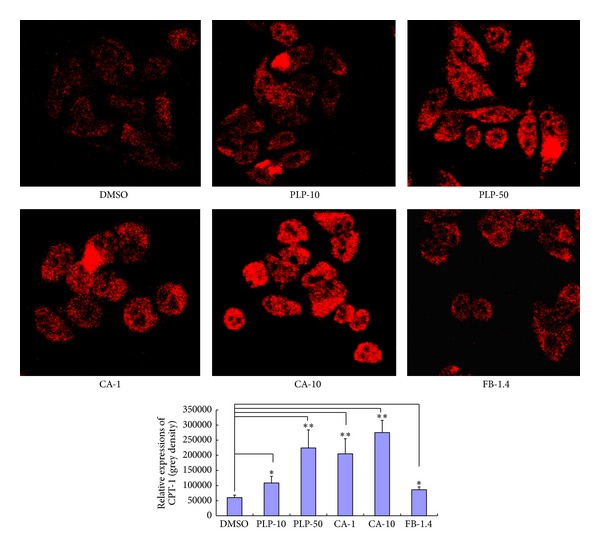
Effect of PLP and CA on CPT-1A expression in HepG2 cells based on the immunofluorescence assay using confocal microscopy. DMSO: DMSO-treated control cells; PLP-10, PLP-50: pineapple leaf phenol-treated (final concentrations of 10 and 50 *μ*g/mL, resp.) cells; CA-1 and CA-10: coumaric acid-treated (final concentrations of 1 and 10 *μ*g/mL, resp.) cells; and FB-1.4: fenofibrate-treated (final concentration of 1.4 *μ*g/mL) cells. Data are expressed as mean ± SD (*n* = 3). ∗*P* < 0.05, ∗∗*P* < 0.01 versus DMSO controls.

**Figure 8:**
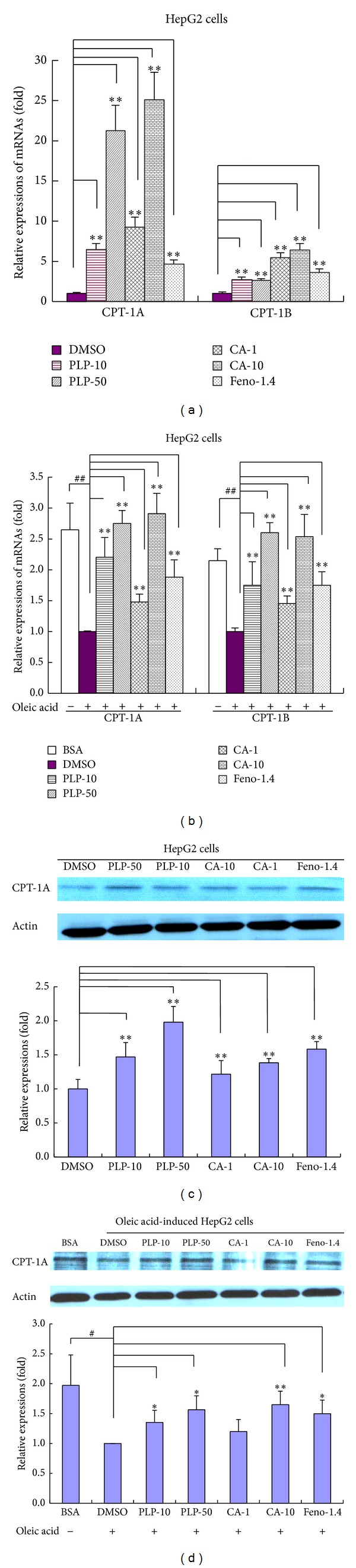
Effect of PLP and CA on CPT-1 expression in oleic acid-untreated ((a) and (c)) and oleic acid-treated ((b) and (d)) HepG2 cells based on RT-PCR and Western blot assays. BSA: bovine serum albumin-treated blank control; DMSO: DMSO-treated control cells; PLP-10, PLP-50: pineapple leaf phenol-treated (final concentrations of 10 and 50 *μ*g/mL, resp.) cells; CA-1 and CA-10: coumaric acid-treated (final concentrations of 1 and 10 *μ*g/mL, resp.) cells; FB-1.4: fenofibrate-treated (final concentration of 1.4 *μ*g/mL) cells; and +/−: incubation with (+) or without (−) oleic acid at the final concentration of 0.6 *μ*M. Data are expressed as mean ± SD (*n* = 3). ^#^ *P* < 0.05, ^##^ *P* < 0.01 versus BSA controls; ∗*P* < 0.05, ∗∗*P* < 0.01 versus DMSO controls.

The immunofluorescence assay showed that PLP (final concentrations of 10 and 50 *μ*g/mL, resp.), CA (final concentrations of 1 and 10 *μ*g/mL, resp.), and FB (final concentration of 1.4 *μ*g/mL) significantly upregulated CPT-1A expression in HepG2 cells (Figure 7). Furthermore, RT-PCR and Western blot assays were used to validate the effects of PLP and CA on CPT-1A expression as shown in the immunofluorescence assay. The results showed that both PLP and CA significantly upregulated CPT-1A mRNA and protein expressions in either oleic acid-treated or oleic acid-untreated HepG2 cells (Figure 8). For mRNA expressions, although the absolute amount of CPT-1B mRNA expression was less than that of CPT-1A (data were not shown), the effects of PLP and CA on CPT-1B and CPT-1A mRNAs were similar. The results of PLP were consistent with the results* in vivo*. However, the effect of FB on CPT-1 expression* in vitro* differed from that* in vivo*. This difference might be due to the fact that the different action times of FB had variable effects on CPT-1 expression (the trials* in vitro* were within 24 h, whereas the trials* in vivo* were about four weeks). Nevertheless, CA was likely an important active component of PLP. However, further trials* in vivo* should be performed in the future to validate these results.

## 4. Discussion

In this study, high-fat diet-fed mice showed a significant increase in abdominal fat and liver lipid accumulation, which was consistent with our previous study [19]. Both PLP and FB significantly attenuated the increase in abdominal fat and liver lipid accumulation in high-fat diet-fed mice. High-fat diet-fed mice had higher calorie intake than normal controls. The increase in calorie intake mainly contributed to the increase in fat accumulation or storage in tissues instead of body weight gain. Despite this effect, the hypolipidemic effects of PLP seemed to have no relationship with dietary calorie intake. FB slightly increased dietary calorie intake but did not contribute to a significant increase in body gain or increase in fat accumulation. Thus, enhanced fat oxidation metabolism may contribute to the effects of PLP.

The liver is one of the main organs for fat oxidation metabolism. The mitochondria and peroxisomes are the main cell organelles responsible for fat oxidation metabolism [20]. Ech1 is a key enzyme that dominates fat oxidation metabolism in peroxisomes [21]. The downregulation of Ech1 contributes to high-fat diet-induced hepatic steatosis [22]. In this study, PLP slightly upregulated the protein expression of Ech1 in HFD mice, but this effect was much weaker than that of FB. By contrast, FB significantly upregulated the mRNA and protein expressions of Ech1. Acox1, Acaa1, and Ehhadh are important genes responsible for fat oxidation in peroxisomes [23]. Higher expression levels of these genes may indicate more fatty acid oxidation metabolism in peroxisomes. In this study, FB promoted the expression of these peroxisome genes, but PLP had no significant effects. These results suggest that peroxisomes could be targeted cell organelles for FB but not for PLP.

CPT-1 is a key rate-limiting enzyme in charge of transporting long-chain fatty acids into mitochondria. The overexpression of CPT-1 in skeletal muscles* in vivo* increases fatty acid oxidation and reduces triacylglycerol esterification [24]. Moreover, the stimulation of systemic CPT-1 activity may be an attractive means to accelerate peripheral fatty acid oxidation [25]. CPT-1A is mainly expressed in livers. CPT-1B is mainly expressed in muscles and also expressed in livers. However, CPT-1B expressed in livers is far less than CPT-1A. Here, PLP significantly promoted the mRNA and protein expression of CPT-1. However, four weeks of FB administration seemed to have no significant effect on the expression of CPT-1* in vivo*. PLP attenuated fat accumulation in the liver, which could be related to the upregulation of CPT-1 expression. Furthermore, PLP induced a greater increase in AMPK and UCP-1 expression than FB. AMPK and UCP-1 are key factors responsible for mitochondrial energy metabolism [26, 27]. These results indicate that PLP acted mainly through targeting mitochondrial functions.

PPAR*α* is a transcription factor and major regulator of lipid metabolism in the liver. The activation of PPAR*α* promotes uptake, utilization, and catabolism of fatty acids by upregulating genes involved in peroxisomal and mitochondrial fatty acid *β*-oxidation [28]. PGC-1*α* is a regulator of mitochondrial biogenesis and function [29]. The activation of PPAR*α* promotes the expression of Ech1 [30], which was consistent with our results. Both PPAR*α* and PGC1*α* stimulated the transcription of the CPT-1A gene [31]. PLP significantly promoted the expression of CPT-1 by upregulating the expression of PPAR*α* and PGC1*α*. However, FB seemed to have no effect on the protein expression of CPT-1 in the livers of mice even though FB activated PPAR*α*. In animal trials, FB activated PPAR*α* for a long period (about four weeks), which possibly triggered the negative regulation of PPAR*α* expression because of the homeostatic responses* in vivo*. These responses may explain why FB decreased the expression of PPAR*α* at a late stage; this decrease could directly affect the regulation of CPT-1 expression. In livers of mice after acute administration of tetradecylglycidic acid, a PPAR*α* ligand, CPT1A, and CPT1B were significantly upregulated [32]. It seemed that the expressions of CPT1A and CPT1B might vary with the time of PPAR*α* activation or might not be completely mediated by PPAR*α* pathway.

CA is one of the main components of PLP [17]. In this study, both PLP and CA showed a significant effect on the attenuation of fat accumulation in oleic acid-induced HepG2 cells. Furthermore, both PLP and CA upregulated the expression of CPT-1 in HepG2 cells. The results of PLP* in vitro* were consistent with those of the animal study* in vivo*. In our previous study, CA demonstrated preferable pharmacokinetic parameters after oral administration in mice [18]. Thus, CA could be considered as a marker component in PLP. However, further investigations* in vivo* should be conducted.

Beside impaired fatty acid beta oxidation, liver fat accumulation can be traced by the increased incidence of* de novo* lipogenesis [33]. In this study, we mainly investigated the effect of PLP and CA on hepatic fat oxidation metabolism. However, the effect of PLP and CA on hepatic lipogenesis might require further investigation in the future. In addition, PLP and CA have strong antioxidant properties against the production of oxidative stress* in vitro* in our preliminary trials (data were not shown). It remains interesting to investigate whether the effects of PLP and CA are associated with the inhibition of prooxidative state induced by HFD* in vivo* and likely by oleic acid treatment* in vitro* in the future since NAFLDs were associated with increased oxidative stress [34].

## 5. Conclusion

Taken together, we firstly found that PLP inhibited the formation of fatty livers and enhanced fat oxidation metabolism in the mitochondria of livers possibly by targeting CPT-1. By contrast, FB mainly targeted the related genes in peroxisomes. CA could be one of the main active components of PLP. PLP likely served as a natural component with novel hypolipidemic mechanisms and may be a promising natural alternative to improve NAFLDs.

## Figures and Tables

**Table 1:** Mitochondrial and peroxisomal *β*-oxidation genes in this study.

Gene names	NCBI accession number	Primers (5′→3′)	Sizes (bp)
Ech1	NM_016772.1	Forward: GGAGGGAGTTGGTGGAAT	291
		Reverse: CACAGGCAGAAACGAGGT	
Acox1	NM_015729	Forward: CCGCCTATGCCTTCCACT	182
		Reverse: ACCGCAAGCCATCCGACA	
Ehhadh	NM_023737.3	Forward: TGGACCATACGGTTAGAG	213
		Reverse: CAATCCGATAGTGACAGC	
Acaa1	NM_130864.3	Forward: GATGACCTCGGAGAATGTGG	188
		Reverse: CCTGAGACACGGTGATGGT	
PPAR*α*	NM_011144	Forward: TACTGCCGTTTTCACAAGTGC	122
		Reverse: AGGTCGTGTTCACAGGTAAGA	
PGC1*α*	NM_008904.2	Forward: ACAGCAAAAGCCACAAAG	259
		Reverse: TAAGGTTCGCTCAATAGTC	
CPT-1A	NM_013495.2	Forward: CGTGACGTTGGACGAATC	165
		Reverse: TCTGCGTTTATGCCTATC	
CPT-1B	NM_009948.2	Forward: GCACACCAGGCAGTAGCTTT	107
		Reverse: CAGGAGTTGATTCCAGACAGGTA	
UCP-1	NM_009463	Forward: AGGCTTCCAGTACCATTAGGT	133
		Reverse: CTGAGTGAGGCAAAGCTGATTT	
AMPK	NM_001013367	Forward: GTCAAAGCCGACCCAATGATA	100
		Reverse: CGTACACGCAAATAATAGGGGTT	
*β*-Actin	NM_007393	Forward: GTGACGTTGACATCCGTAAAGA	245
		Reverse: GCCGGACTCATCGTACTCC	

## Abbreviations

Acaa1: 3-Ketoacyl-CoA thiolase
Acox1: Acyl-CoAoxidase 1 palmitoyl
AMPK: Adenosine 5′-monophosphate (AMP)-activated protein kinase
CA: p-Coumaric acid
CPT-1: Carnitine palmitoyltransferase 1
DMSO: Dimethyl sulfoxide
Ech1: Enoyl coenzyme A hydratase 1, peroxisomal
Ehhadh: Enoyl-CoA hydratase/*L*-3-hydroxyacyl-CoA dehydrogenase
FB: Fenofibrate
HFD: High-fat diets
NAFLDs: Nonalcoholic fatty liver diseases
PGC1*α*: Peroxisomal proliferator-activated receptor gamma coactivator *α*
PLPs: Pineapple leaf phenols
PPAR*α*: Peroxisomal proliferator-activated receptor alpha
UCP-1: Uncoupling protein 1.
